# Variation in Seeking Care for Cardiovascular Disease and Ambulance Utilization among Migrants in Australia: Time, Ethnicity, and Delay (TED) Study III

**DOI:** 10.3390/ijerph19031516

**Published:** 2022-01-28

**Authors:** Kannikar Hannah Wechkunanukul, Shahid Ullah, Justin Beilby

**Affiliations:** 1 Public Health Department, Torrens University Australia, Adelaide, SA 5000, Australia; 2 College of Nursing and Health Sciences, Flinders University, Bedford Park, SA 5042, Australia; 3 College of Medicine and Public Health, Flinders University, Bedford Park, SA 5042, Australia; shahid.ullah@flinders.edu.au; 4 Academic Research, Torrens University Australia, Adelaide, SA 5000, Australia; jbeilby@Torrens.edu.au

**Keywords:** chest pain, migrant, culturally and linguistically diverse, ethnicity, delay time, decision time, prehospital delay, seeking medical care, ambulance utilization

## Abstract

Insight into differences in seeking medical care for chest pain among migrant populations is limited. This study aimed to determine ethnic differences in seeking care behaviors and using ambulances among migrants compared to an Australian-born group. A total of 607 patients presenting with chest pain to a tertiary hospital between 1 July 2012 and 30 June 2014 were randomly selected. Data from the emergency department dataset and medical record reviews were collected and linked for analysis. The migrant group was stratified into nine ethnic groups for analysis based on the Australian Standard Classification of Cultural and Ethnic Groups. The overall median prehospital delay time was 3.7 (1.5, 10.7) h, which ranged from 2.5 (1.0, 10.7) (Southern and Eastern European group) to 6.0 (2.3, 20.6) (Sub-Saharan African group). The median decision time was 2.0 (0.8, 7.9) h, which ranged from 1.5 (Australian-born group) to 4.5 h (Sub-Saharan African group). Five ethnic groups had significantly longer decision times compared to the Australian-born group. Decision time accounted for 58.4% of pre-hospital delay time. Migrant patients were 60% less likely to seek care for chest pain within one hour (odds ratio 0.40, (0.23–0.68), *p* = 0.001). There was no significant difference in ambulance utilization between migrant and Australian-born groups. In conclusion, ethnic differences in seeking care for chest pain do exist, and ethnicity plays a vital role in a longer delay in seeking care. To reduce the delays and improve patient outcomes, appropriate health campaigns focusing on ethnic differences among migrant populations and normalizing cultural competency into practice are recommended.

## 1. Introduction

Globally, pre-hospital delay times have varied from country to country, even between countries in the same continent [1]. McKee, et al. [2] concluded that behavioral factors and symptom presentations contributed to predictive pre-hospital delay. The cognitive and emotional response of the patient, and psychological and social factors were also found to be associated with longer delay times [3,4], as were ethnicity and cultural factors [5,6]. Variations in characteristics (e.g., age, education level, socioeconomic status), symptoms (e.g., symptom recognition, perception of pain, severity of pain), and outcomes (e.g., mortality rate, readmission rate) among ethnic groups have been reported in previous studies [6,7,8]. Rye et al. [9] reported a higher risk of cardiovascular and comorbidity among migrants compared to Australian-born patients [9]. One study revealed a low level of awareness of myocardial infarction (MI) symptoms and inappropriate response to MI symptoms among migrants in the USA [10].

A recent study noted that atypical symptoms of acute coronary syndrome were commonly reported for South Asian and Chinese patients. They also found that Asian migrants took a longer time to present to the emergency department (ED) [11]. Consistently, a systematic review reported the association between ethnicity and delay time in seeking medical care for chest pain where migrants delayed longer than the majority population [12]. King-Shier et al. [13] concluded that ethnic or cultural differences had an impact on how migrants navigate the healthcare system. The delay in seeking medical care for acute MI among migrants and women were also reported during the COVID-19 pandemic due to fears of being infected in hospitals [14].

Although sub-analyses for migrant populations by ethnic groups have been conducted in some multicultural countries (e.g., Canada [6], Sweden [15], the UK [5], and the USA [16]), only some ethnic groups were included based on the limitations of each study such as a small percentage of ethnic groups and ethnic classification methods. The exclusion of migrants or non-English speaking patients could influence the research findings [17,18]. Differences in composition of ethnic participants between studies may have led to such varied research outcomes. As a multicultural society, one-third of the Australian population are migrants or culturally and linguistically diverse (CALD) people [19]. Nonetheless, there was a limited database of care-seeking behaviors among all ethnic groups who migrated to Australia. It is important to determine the differences in seeking care behaviors in migrant populations to understand their unique problems and needs. Therefore, further research to investigate in ethnic groups among migrant population is warranted [20].

The Time, Ethnicity, and Delay (TED) study is a triangulation involving a systematic review (TED I) [12], a cross-sectional analysis of an emergency department cohort (TED II) [21], and this study, a retrospective medical record review (TED III) to investigate delay in seeking medical care for chest pain among migrant populations.

The aim of TED III was to determine the differences in seeking medical care for chest pain between migrants from 74 countries within nine ethnic groups compared to Australian-born patients. This study focuses on the differences in decision time, pre-hospital delay, ambulance utilization, and the influence of ethnicity on responding to chest pain.

The specific objectives of this study are as follows:

1. To compare decision time and pre-hospital delay time between migrant and the Australian-born groups,
2. To determine differences in delay times between the nine ethnic groups within the migrant group;
3. To examine the influence of ethnicity on decision time to seek care for chest pain;
4. To compare ambulance utilization rate between migrant and the Australian-born groups.

## 2. Materials and Methods

### 2.1. Study Design and Setting 

This retrospective study extracted data from an Emergency Department Information System (EDIS) dataset and the medical records of patients presenting with chest pain at the Emergency Department (ED) of a metropolitan public hospital in South Australia. The Department of Cardiovascular Medicine of this setting provides specialist cardiac care services including a chest pain assessment unit, angiography, percutaneous interventions, and coronary artery bypass grafting (CABG). Annually, there are 75,000 ED visits, and 5.5% of all visits present with chest pain. One-third of all chest pain cases are migrant patients who were born outside Australia based on their self-identification.

### 2.2. Participants

#### 2.2.1. Inclusion and Exclusion Criteria

The patients who presented to the ED between 1 July 2012 and 30 June 2014 were identified for eligibility. The inclusion criteria were (1) chest pain reported as a chief complaint; (2) country of birth was recorded; and (3) time of arrival (triage time) was recorded. 

The exclusion criteria were (1) country of birth was unclassified; and (2) medical record was not available.

#### 2.2.2. Sample Selection and Study Size

Stratified random sampling was employed to obtain cases using a computer program to generate random lists for medical records review. A stratified design, which divided the sample among nine strata (ethnic groups), was analyzed using the two-sided, Cochran–Mantel–Haenszel test to reject the odds ratio (OR). A sample size was based on a previous study [22] that evaluated the effects of whether African-Americans delayed longer than non-Hispanic Whites during an acute myocardial infarction. African-American participants were less likely (OR = 0.34) to access the emergency medical service (EMS) during the critical first hour after the onset of symptoms. As the confidence interval was high (95% CI 0.14–0.85) in their study, we chose to be conservative in our estimates by choosing the middle of the CI (OR = 0.495) to increase the likelihood of achieving the highest statistical power possible. Assuming an alpha error of 0.05 and a beta error of 20%, power analysis indicated that n = 552 participants would be required (N1 = 276 in migrant group and N2 = 276 in Australian-born group) to reach at least 80% power at a 5% level of significance.

### 2.3. Data Source and Data Collection

The data for this study were extracted from two datasets. The basic demographic information and ED functional information of presenting patients were derived from the EDIS. The further variables (medical history, time variables, social and cultural variables, and clinical outcomes) were extracted from medical records. The medical records were reviewed by the first reviewer using a structured review form, and the second opinion was sought from the second reviewer if necessary. A third opinion was sought if any conflicting opinions occurred between the first two reviewers.

### 2.4. Definitions

1. Australian refers to patients whose country of birth was classified as Australia.
2. Migrants refers to patients whose country of birth was classified as a country other than Australia.
3. Country of birth in this study refers to self-identification on country of birth by individual patients, which was recorded in the EDIS and medical records.
4. Ethnicity was classified by country of birth using the Australian Standard Classification of Cultural and Ethnic Groups [23]. The classification of countries and ethnic groups is available in Table 1.
5. Culturally and linguistically diverse (CALD) population was defined as individuals born overseas in a country other than countries classified as a ‘main English country’ by the Australian Bureau of Statistics, speak a language other than English, and may speak English as a second language, including people from English- speaking backgrounds whose culture, ethnicity, religion, and spirituality background differ from those of the Australian mainstream [24,25].

### 2.5. Variables and Data Measurements

The presenting characteristic variables were collected from the EDIS and the medical records. The migrant group was stratified into nine groups, including (1) Oceanian; (2) North-West European; (3) Southern and Eastern European; (4) North African and Middle Eastern; (5) South-East Asian; (6) North-East Asian; (7) Southern and Central Asian; (8) People of the Americas; and (9) Sub-Saharan African, based on the Australian Standard Classification of Cultural and Ethnic Groups [23]. 

Decision time was defined as the amount of time between the time of symptom onset to accessing the emergency response system or to initiating travel to the hospital [26]. The cut-off point of one hour for decision times was chosen based on the guidelines for the management of acute coronary syndromes and from previous studies [27,28]. Prehospital delay time was defined as the amount of time between the time of symptom onset and hospital arrival [26,29].

### 2.6. Statistical Analysis

The data were analyzed using IBM SPSS Statistics Version 25.0. (IBM Corp. Armonk, New York, NY, USA). The *p*-values for all statistical significance were set at <0.05. The patient demographics and presentations were stratified by migrant groups and the Australian-born group. Categorical variables were described as frequencies and percentages, and a Chi-square test was used for the comparisons. Continuous variables with normal distribution were described as a mean with standard deviation (SD), and an independent sample *t*-test was used for comparisons. For the skewed distribution, the median (25th, 75th percentile) was presented and compared by using the Mann–Whitney U-Test. The two logistic regression models were used to determine the independent predictors of decision time within one hour, and ambulance was used as the first medical contact. The presenting characteristics (22 variables) were placed into the regression models.

## 3. Results

**Figure 1:**
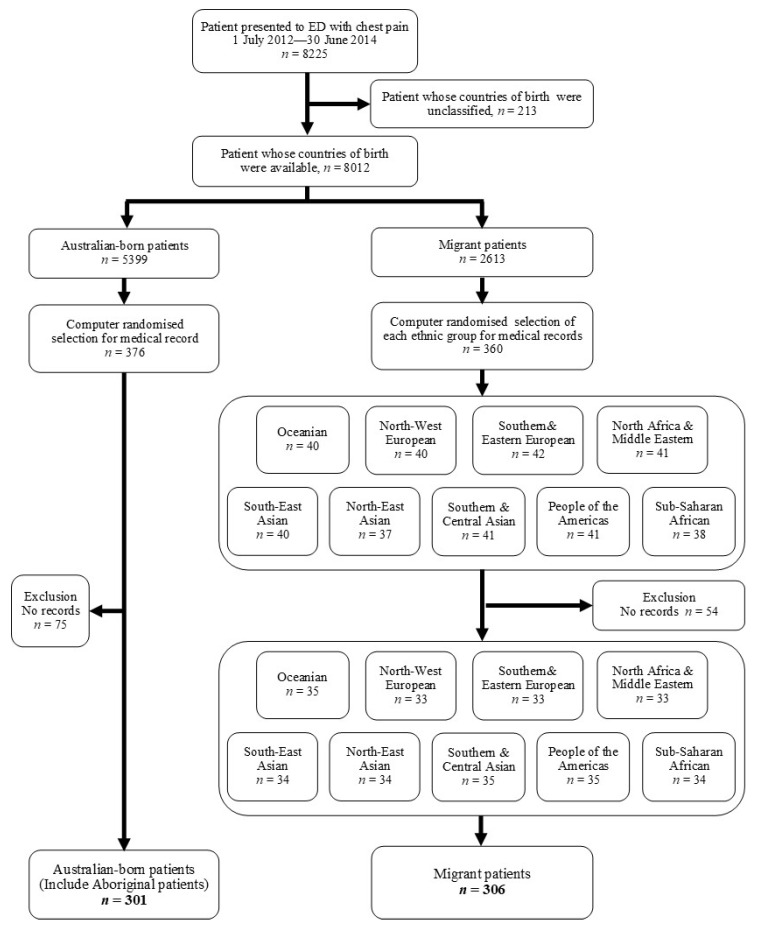
Study flow diagram.

A total of 8225 patients with chest pain presented to the ED between 1 July 2012 and 30 June 2014 were assessed for eligibility. The 213 patients were first excluded due to unclassified country of birth, leaving 8012 patients included in the next step. All patients were allocated into two groups; 2613 (32.6%) migrant patients and 5399 (67.4%) Australian-born patients, based on their countries of birth. Then, all ethnic patients from 74 countries were stratified into nine ethnic groups based on the Australian Standard Classification of Cultural and Ethnic groups (Table 1). Patients in each group were randomly selected, including 360 patients from the migrant group and 376 patients from the Australian-born group. Due to a lack of medical records, 54 migrant patients and 75 Australian-born patients were excluded. Finally, 306 patients from the migrant groups and 301 patients from the Australian-born group were included in the study, resulting in 607 patients being eligible for medical record reviews (Figure 1).

### 3.1. Presenting Characteristics

The mean age ± SD for all patients was 56 ± 19.1 years and ranged between 46 ± 17.7 years (Southern and Central Asian) and 70 ± 16.1 years (Southern and Eastern European). The Southern and Eastern European and North-West European groups were significantly older than the Australian-born group (mean age ± SD: 70 ± 16.1 vs. 55 ± 19.9, *p* < 0.001; and 68 ± 17.5 vs. 55 ± 19.9, *p* = 0.001, respectively). Sub-Saharan Africans and Southern and Central Asians were significantly younger than Australian patients (mean age ± SD: 48 ± 14.4 vs. 55 ± 19.9 *p* = 0.016; and 46 ± 17.7 vs. 55 ± 19.9, *p* = 0.010, respectively) (Table 2). Males made up just over half of the population at 51.4%, and 44.5% of the entire population reported married status.

All Australian and European patients were covered by Medicare (Universal Health Coverage). By contrast, the remaining ethnic groups had a significantly lower proportion of eligibility for Medicare compared to those former three groups, ranging from 73.5% (North-East Asian) to 97.1% (Oceanian, South-East Asian, and the Americas). Language barriers reported by health professionals were occurred among all ethnic groups (except for the North-West European group), ranging from 2.9% (Oceanian) to 41.2% (North-East Asian) (Table 2).

### 3.2. Ethnic Differences in Seeking Care for Chest Pain

#### 3.2.1. Comparisons of Decision Times

**Figure 2:**
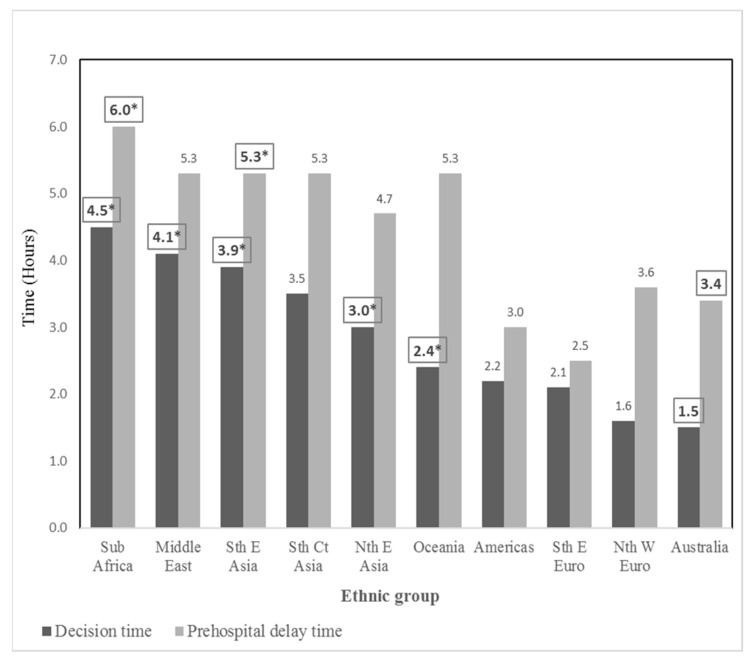
Comparisons of decision time (hours) and pre-hospital delay time (hours) between ethnic and Australian-born groups. Nth W Euro: North-West European, Sth E Euro: Southern and Eastern European, Nth E Asia: North-East Asian, Sth E Asia: South-East Asian, Sth Ct Asia: Southern and Central Asian, Middle East: North African and Middle Eastern, Americas: People of the Americas, Sub Africa: Sub-Saharan African. Decision time was defined as the interval from the time of symptom onset to accessing the emergency response system or to initiating travel to the hospital [26]. Prehospital delay time was defined as the interval between the time of symptom onset and hospital arrival [26,29]. * Significant differences between Australian and ethnic groups, *p* < 0.05 by Mann–Whitney U test.

The median (25th, 75th percentile) decision time (hours) for this study was 2.0 (0.8, 7.9) and ranged from 1.5 (Australian) to 4.5 (Sub-Saharan African). There were five ethnic groups that had significantly longer decision times compared to the Australian-born group: the Sub-Saharan African group; the North African and Middle Eastern group; the South-East Asian group; the North-East Asian group; and the Oceanian group. Details are presented in Table 3 and Figure 2. There were four statistically significant differences in decision time among the nine ethnic groups: the North-West European and the South-East Asian groups (1.6 (0.8, 5.7) vs. 3.9 (2.2, 21.3), *p* = 0.009); the North-West European and the Sub-Saharan African groups (1.6 (0.8, 5.7) vs. 4.5 (1.8, 14.3), *p* = 0.025); the Southern and Eastern European and the South-East Asian groups (2.1 (0.5, 8.5) vs. 3.9 (2.2, 21.3), *p* = 0.012); and the Southern and Eastern European and the Sub-Saharan African groups (2.1 (0.5, 8.5) vs. 4.5 (1.8, 14.3), *p* = 0.035).

**Figure 3:**
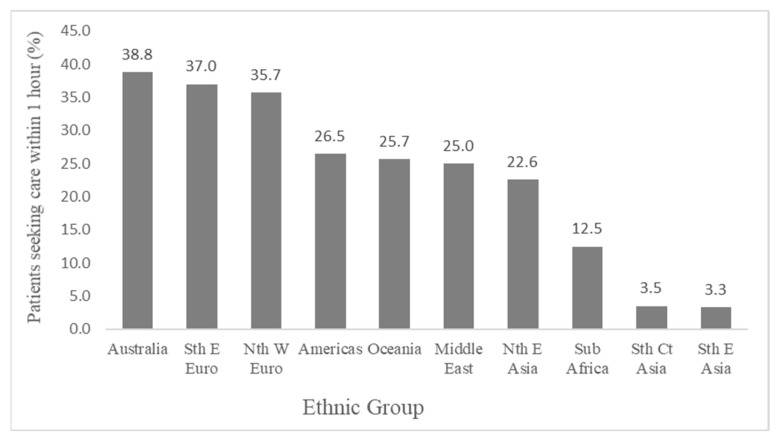
Comparisons of proportion of patients seeking care for chest pain within one hour between ethnic and Australian-born groups.

Overall, 182 (31.9%) patients initially sought for medical care within one hour when experiencing chest pain. The proportion of patients making a decision within 1 h ranged from 3.3% (South-East Asian) to 38.8% (Australian) (Figure 3). The breakdown of decision time into time categories (hours) is presented and compared in Table 3.

#### 3.2.2. Comparisons of Prehospital Delay Time

In total, the median (25th, 75th percentile) pre-hospital delay time (hours) was 3.7 (1.5, 10.7), ranging between 2.5 (1.0, 10.7) (Southern and Eastern European) and 6.0 (2.3, 20.6) (Sub-Saharan African). The median pre-hospital delay times of two ethnic groups were significantly longer than those of the Australian-born group, including the Sub-Saharan African (6.0 (2.3, 20.6) vs. 3.2 (1.4, 8.8) h, *p* = 0.025) and the South-East Asian (5.3 (3.0, 22.3) vs. 3.2 (1.4, 8.8) h, *p* = 0.012) (Table 3). There were two statistically significant differences in pre-hospital delay time among nine ethnic groups: the Southern and Eastern European and the South-East Asian (2.5 (1.0, 10.7) vs. 5.3 (3.0, 22.3), respectively, *p* = 0.011); and the Southern and Eastern European, and the Sub-Saharan African (2.5 (1.0, 10.7) vs. 6.0 (2.3, 20.6), respectively, *p* = 0.028). Overall, the contribution of decision time to pre-hospital delay for the entire patients was 58.4%, ranging from 48.5% (Australian) to 83.2% (South-East Asian). The differences in decision time contributions between ethnic groups are presented in Table 3.

#### 3.2.3. Independent Predictors of Decision Time within One Hour

The results of the binary logistic regression analysis of decision time within one hour are available in Table 4. Adjusting for socio-demographics, presenting, and cultural factors, the migrant patients were 60% (95% CI, 0.23, 0.68, *p* = 0.001) less likely than the Australian patients to seek medical care within one hour when experiencing chest pain. There were three other independent predictors of decision time within one hour, including being male, symptom onset during nighttime (18.01–5.59), and having chest pain during activities. Patients who experienced chest pain at nighttime (18.01–5.59) were 2.19 times (95% CI, 1.27, 3.77, *p* = 0.005) more likely to seek medical care within one hour than those who experienced chest pain during the daytime (6.00–18.00). Having onset of chest pain during activities increased the chance of seeking medical care within one hour by 1.88 times (95% CI, 1.02, 3.50, *p* = 0.045) in comparison to onset taking place during resting position. Male patients were 1.79 times (95% CI, 1.05, 3.07, *p* = 0.034) more likely than female to make their decision within one hour.

### 3.3. Ambulance Utilization and Its Independent Predictors

The rate of ambulance utilization as the first medical contact when patients experienced chest pain for all patients was 35.7%. The proportion of patients calling an ambulance varied across the ethnic groups, ranging from 22.9% (Southern and Central Asian) to 54.4% (North-West European), but this was not statistically significant difference (Table 2).

Adjusting for socio-demographic, medical history, and presenting factors, migrant status was not associated with ambulance utilization. Age, alcohol consumption history, prior stroke/TIA, symptom onset at nighttime, and pain score significantly contributed to the prediction of ambulance utilization (Table 5). Patients with prior history of stroke/TIA were 7.39 times (95% CI, 1.24, 40.3, *p* = 0.021) more likely to call an ambulance upon experiencing chest pain. The cardiac event occurring at nighttime (18.01–5.59) increased the chance of calling an ambulance by 2.68 times (95% CI, 1.54, 4.66, *p* < 0.001) more than the event occurring during the daytime (6.00–18.00). Patients were more likely to call an ambulance 1.13 times (95% CI, 1.04, 1.24, *p* = 0.007) for every additional scale of pain score. Patients were more likely to arrive at the hospital by ambulance 1.04 times (95% CI, 1.02, 1.06, *p* < 0.001) for every additional year of age. The history of alcohol consumption decreased the chance of calling an ambulance when experiencing chest pain by 51% (95% CI, 0.27, 0.90, *p* = 0.021) compared to those with a non-alcohol history.

## 4. Discussion

### 4.1. Presenting Characteristics of Samples

Generally, characteristics of the 607 patients in the TED study III were comparable to the cohort of 8012 patients of its primary study [21]. The significantly lower rate of accessibility to Medicare (universal health coverage) among seven ethnic groups (except for the two European groups) compared to the Australian-born group might be related to the waiting period (104 weeks) before being eligible to access Medicare for newly arrived migrants [30]. By contrast, many European countries, such as the United Kingdom, Sweden, Finland, Italy, Belgium, Malta, and Slovenia are eligible to access essential medical treatment during visiting Australia through the Reciprocal Health Care Agreements with Australia [31]. This could be the explanation of the equal rate of Medicare accessibility between the European and the Australian-born patients in this study which is consistent with the finding of previous study concluding that health care insurance caused differences in access to primary care between immigrants and non-immigrants [32].

### 4.2. Ethnic Differences in Seeking Care for Chest Pain

The median decision time of 2.0 h indicates an unchanged level of patient delay time comparing to a previous Australian study [33] and an international study [34] conducted in the last decade. Patients from Africa, Middle East, and Asia regions had the longest delays, taking more than five hours to reach the hospital, while the Australians and Europeans had the shortest delays. However, all groups did not reach the ED within one hour to achieve optimal outcomes from the definitive treatment, which should be initiated within one hour [35]. These findings were consistent with previous multi-ethnic studies in the USA [36] and Canada [6], where all ethnic and dominant patients delayed longer than one hour to seek medical care for chest pain. This study found the comparable results with Zerwic, Ryan, DeVon, and Drell [22] revealing that ethnicity was a significant predictor of responding to chest pain within one hour after the symptom onset. Therefore, a further investigation on a large scale for each ethnic group is recommended to explain their seeking care behavior. 

The majority (58.4%) of the entire pre-hospital delay time was attributed to decision time, which is comparable to other Australian studies [37,38] and international studies [34,39]. Symptom recognition and the cognitive and emotional responses of the patients have been found to influence decision times [3,40]. Many researchers suggested that improving decision times can help in reducing total pre-hospital delays [3,34,39]. Additionally, Richards et al. [41] concluded that perceptions of symptoms and illness behaviors were shaped by the patients’ cultural and social contexts. Therefore, a culturally sensitive investigation of ethnic groups which emphasizes the impact of culture and ethnicity on different seeking care behavior is recommended [13,28,42,43].

### 4.3. Ambulance Utilization and the Predictors

The ambulance utilization rate of 35.7% in this study was comparable to its primary study [21] and previous Australian study [44]. In Australia, emergency ambulance services charges (approximately $500–$1300.00/time for emergency call) are not covered by Medicare or basic private health insurance [45], but individuals have to pay for the ambulance cover separately. The lack of ambulance cover and cost of services may possibly be taken into account when patients are considering whether to call the ambulance. Meischke et al. [46] noted that one of the main reasons for not calling an ambulance was that the ‘symptoms were not severe’. The findings in TED III found the similar result that a higher degree of pain contributed to a higher chance of calling an ambulance. Two studies reported that media campaigns did not increase ambulance utilization [44,47]. Another study concluded that the post-public campaign rate of ambulance usage tapered off over time [48]. Strategies for encouraging people to use an ambulance when experiencing chest pain require future interventions and appropriate initiatives to increase and sustain access to emergency services. The long-term and ongoing action health strategies for increasing ambulance utilization as the first contact should be focused rather than short-term solutions [49].

### 4.4. Implications for Research and Practice

The Australian government has provided national guidelines and programs to promote equity in accessing healthcare, including Multicultural Language Services Guidelines for Australian Government Agencies [50] and Multicultural Service Officers [51], which enable non-English speaking patients to access care. However, a longer decision time and pre-hospital delay among ethnic groups related to cultural and language barriers still exist in Australia. The evaluation of the effectiveness of these national programs should be considered, particularly at the community level.

Cultural and social factors should be incorporated into public campaigns and health promotions. Cultural competence and cultural safety should be normalized into the healthcare system to help improving care for and understanding the unique problems and needs of specific ethnic groups [13,52]. The current delay in presentation for migrant patients is a real concern. It is important that information and education campaigns should target key health care providers, including general practitioners, nurse practitioners, pharmacists, community healthcare workers, refugee health services, and other key community leaders. These cultural community linked practitioners are important influencers in patient health behavior. For patients at risk of cardiac events, a delay in presentation for chest pain and accessing ambulance services must be part of the normal day-to-day consultation.

The translation of education resources into languages other than English at the individual and community levels such as web-based application, mobile phone applications, wearable device, radio programs, and community television programs can help patients access healthcare information and healthcare services. Additionally, using interpreters in community education may help with improving accessibility in healthcare information among migrant populations. Digital health interventions for cultural groups should be considered to address health system challenges among this disadvantaged population at the individual level (e.g., low adherence, insufficient engagement, unaware of services, ineffective communication); community levels (e.g., different perception of disease and care, lack of feedback mechanisms, lack of alignment with social norm); and system (e.g., lack of cultural competency, insufficient workforce, delay in provision of care, poor referrals) [53].

### 4.5. Limitations

There are some limitations of TED III. Firstly, obtaining a dataset from a single hospital may be inappropriate for generalizing the findings to the entire population. However, the samples were randomly selected from a large population (n = 8012) that reflected national population proportions. This study included a small sample size from nine ethnic groups due to the time limit for conducting the study, but the sample sizes were achieved over 80% of the power analysis. Finally, TED III is a retrospective study that may have inherent biases in data selection, collection, and analysis, such as missing data [54]; nonetheless, TED III has a only a small percentage of missing data (5.9%) due to a lack of symptom onset.

## 5. Conclusions

The average decision times and prehospital delay times of all groups including the Australian-born group were greater than the recommended timeframe of one hour. This study found that ethnic differences in seeking care for chest pain do exist, and migrant status is a significant predictor of patient delay. These messages will be essential information for health agencies and researchers working with ethnic populations. Future interventions, including digital health innovation and education campaigns focusing on ethnic differences are recommended. Lastly, it is warranted that cultural competence should be normalized into the healthcare system to reduce health inequities and increase accessibility.

## Figures and Tables

**Table 1:** Classification of countries and ethnic groups based on the Australian Standard Classification of Cultural and Ethnic groups.

Group	Ethnic Group	Narrow Group	Country
1	Oceanian	Australian Peoples New Zealand Peoples Melanesian and Papuan Micronesian Polynesian	Australia, Australian Antarctic territory New Zealand Solomon, Papua New Guinea Fiji
2	North-West European	British Irish Western European Northern European	England, Scotland, the UK Ireland, Northern Ireland German, Netherlands
3	Southern and East European	Southern European South-Eastern European Eastern European	Italy, Malta Bulgaria, Croatia, Cyprus, Greece, Kosovo, Macedonia, Serbia Latvia, Poland, Russian, Ukraine
4	North African and Middle Eastern	Arab Jewish Other North African and Middle Eastern	Egypt, Iraq, Lebanon, Saudi Arabia, Syria Israel Iran, Sudan Turkish
5	South-East Asian	Mainland South-East Asian Maritime South-East Asian	Cambodia, Thailand, Vietnam Indonesia, Malaysia, Philippines, Singapore
6	North-East Asian	Chinese Asian Other North-East Asian	China, Hong Kong, Japan, South Korea, Mongolia
7	Southern and Central Asian	Southern Asian Central Asian	Bangladesh, India, Pakistan, Sri Lanka
8	People of the Americas	North Americas South American Central American Caribbean Islander	Canada, The USA Argentina, Brazil, Chili, Columbia, Ecuador, Guyana, South America (NFD), Uruguay El Salvador
9	Sub-Saharan African	Central and West Africa Southern and East Africa	Cameroon, Cote D’Ivoire, Nigeria, Senegal, Sierra Leone Eritrea, Kenya, Mauritius, South Africa, Southern and East Africa, Zambia, Zimbabwe

**Table 2:** Presenting characteristics of patients presenting to emergency department with chest pain by ethnic groups.

Characteristics	Australian *n* = 301	Nth W Euro *n* = 33	Sth E Euro *n* = 33	Nth E Asia *n* = 34	Sth E Asia *n* = 34	Sth Ct Asia *n* = 35	Middle East *n* = 33	Sth Africa *n* = 34	Americas *n* = 35	Oceanian *n* = 35
Age (years), mean ± SD	55 ± 19.9	68 ± 17.5 *↑	70 ± 16.1 *↑	52 ± 18.3	58 ± 18.4	46 ± 17.7 *↓	57 ± 17.3	48 ± 14.4 *↓	50 ± 17.2	58 ± 12.8
Male, n (%)	163 (54.2)	20 (60.6)	19 (57.6)	13 (38.2)	11 (32.4) *↓	19 (54.3)	18 (54.5)	16 (47.1)	18 (51.4)	15 (42.9)
Medicare, n (%)	301 (100.0)	33 (100.0)	33 (100.0)	25 (73.5) **↓	33 (97.1) **↓	31 (88.6) **↓	29 (87.9) **↓	32 (94.1) **↓	34 (97.1) **↓	34 (97.1) **↓
Language barrier, n (%)	0 (0.0)	0 (0.0)	5 (15.2) *↑	14 (41.2) **↑	4 (11.8) **↑	3 (8.6) **↑	9 (27.3) **↑	2 (5.9) **↑	3 (8.6) **↑	1 (2.9)
Location at home, n (%)	194 (64.5)	27 (81.8) **↑	30 (90.9) **↑	22 (64.7)	24 (70.6)	22 (62.9)	28 (84.8) **↑	24 (70.6)	25 (71.4)	28 (80.0)
First medical contact, n (%)										
Ambulance	116 (38.5)	18 (54.5)	17 (51.5)	8 (23.5)	10 (29.4)	8 (22.9)	9 (27.3)	8 (23.5)	11 (31.4)	12 (34.3)
Emergency department	153 (50.8)	9 (27.3) **↓	11 (33.3)	19 (55.9)	12 (35.3)	16 (45.7)	18 (54.5)	17 (50.0)	20 (57.1)	17 (48.6)
General practitioner	32 (10.6)	6 (18.2)	5 (15.2)	7 (20.6)	12(35.3) **↑	11(31.4) **↑	6 (18.2)	9(26.5) **↑	4 (11.4)	6 (17.1)
Hospital discharged with cardiac diagnosis, n (%)	154 (51.2)	22 (66.7)	21 (63.6)	21 (61.8)	16 (47.1)	17 (48.6)	20 (60.6)	16 (47.1)	17 (48.6)	23 (65.7)

Nth W Euro: North-West European, Sth E Euro: Southern and Eastern European, Nth E Asia: North-East Asian, Sth E Asia: South-East Asian, Sth Ct Asia: Southern and Central Asian, Middle East: North African and Middle Eastern, Sub Africa: Sub-Saharan African, SD: standard deviation, * Significant differences between Australian and ethnic groups, *p* < 0.05 by independent T- test, ** Significant differences between Australian and ethnic groups, *p* < 0.05 by Chi-square test, ↑ more than those of Australian group, ↓ less than those of Australian group.

**Table 3:** Comparisons of decision times and prehospital delay times between ethnic groups.

Delay Time (Hours)	Australian *n* = 289	Nth W Euro *n* = 28	Sth E Euro *n* = 27	Nth E Asia *n* = 31	Sth E Asia *n* = 30	Sth Ct Asia *n* = 33	Middle East *n* = 32	Sth Africa *n* = 32	Americas *n* = 34	Oceania *n* = 35
Decision time, median (25th, 75th percentile)	1.5 (0.5, 4.6)	1.6 (0.8, 5.7)	2.1 (0.5, 8.5)	3.0 *↑ (1.6, 12.1)	3.9 *↑ (2.2, 21.3)	3.5 (0.7, 15.1)	4.1 *↑ (1.0, 22.0)	4.5 *↑ (1.8, 14.3)	2.2 (0.8, 15.4)	2.4 *↑ (1.0, 7.0)
Decision time, n (%)										
≤1	114 (39.4)	10 (35.7)	10 (37.0)	7 (22.6)	1 (3.3) **↓	10 (30.3)	8 (25.0)	4 (12.5) *↓	9 (26.5)	9 (25.7)
1.01–2.00	59 (20.4)	5 (17.9)	3 (11.1)	4 (12.9)	6 (20.0)	4 (12.1)	3 (9.4)	6 (18.8)	7 (20.6)	6 (17.1)
2.01–4.00	39 (13.5)	3 (10.7)	5 (18.5)	5 (16.1)	8 (26.7)	3 (9.1)	5 (15.6)	4 (12.5)	3 (8.8)	5 (14.3)
4.01–8.00	23 (8.0)	5 (17.9)	2 (7.4)	6 (19.4) **↑	5 (16.7)	4 (12.1)	4 (12.5)	8 (25.0) **↑	3 (8.8)	7 (20.0)
>8.00	56 (19.4)	5 (17.9	7 (25.9)	9 (29.0)	10 (33.3)	12 (36.4) **↑	12 (37.5) **↑	10 (31.3)	12 (35.3) **↑	8 (22.9)
Pre-hospital delay time, median (25th, 75th percentile)	3.4 (1.4, 9.0)	3.6 (1.3, 9.9)	2.5 (1.0, 10.7)	4.7 (1.7, 12.3)	5.3 *↑ (3.0, 22.3)	5.3 (1.6, 10.7)	5.3 (1.7, 25.0)	6.0 *↑ (2.3, 20.6)	3.0 (1.2, 15.4)	5.3 (1.6, 14.9)
Proportion of decision time in entire prehospital delay time (%)	48.5	60.7	62.0	74.3 **↑	83.2 **↑	75.8 **↑	64.7 **↑	71.8 **↑	61.8	64.5 **↑

Nth W Euro: North-West European, Sth E Euro: Southern and Eastern European, Nth E Asia: North-East Asian, Sth E Asia: South-East Asian, Sth Ct Asia: Southern and Central Asian, Middle East: North African and Middle Eastern, Sub Africa: Sub-Saharan African, * Significant differences between Australian and ethnic groups, *p* < 0.05 by Mann–Whitney U-test, ** Significant differences between Australian and ethnic groups, *p* < 0.05 by Chi-square test, ↑ more than those of Australian group, ↓ less than those of Australian group.

**Table 4:** Independent predictors of decision time within 1 h.

Predictor	Odds Ratio	95% Confidence Interval	*p*
Socio-demographic factor			
Male	1.79	1.05, 3.07	0.034
Presenting factor			
Symptom onset during nighttime (18.01–5.59)	2.19	1.27, 3.77	0.005
Having active activity during event	1.88	1.02, 3.50	0.045
Cultural factor			
Migrant status	0.40	0.23, 0.68	0.001

Significant at *p* < 0.05.

**Table 5:** Independent predictors of ambulance utilization during the cardiac events.

Predictor	Odds Ratio	95% Confidence Interval	*p*
Socio-demographic factor			
Age	1.04	1.02, 1.06	<0.001
Medical history factors			
Alcohol consumption history	0.49	0.27, 0.90	0.021
Prior stroke/transient ischemic attack	7.39	1.24, 40.3	0.021
Presenting factor			
Symptom onset during nighttime (18.01–5.59)	2.68	1.54, 4.66	<0.001
Pain score	1.13	1.04, 1.24	0.007

## Data Availability

The data presented in this study are available on request from the corresponding author. The data are not publicly available due to ethical restrictions.
